# The heterojunction effects of TiO_2 _nanotubes fabricated by atomic layer deposition on photocarrier transportation direction

**DOI:** 10.1186/1556-276X-7-231

**Published:** 2012-04-23

**Authors:** Yung-Huang Chang, Chien-Min Liu, Chih Chen, Hsyi-En Cheng

**Affiliations:** 1 Department of Materials Science and Engineering, National Chiao Tung University, Hsin-chu, Taiwan, 30010, Republic of China; 2 Department of Electro-optical engineering, Southern Taiwan University, Tainan, Taiwan, 710, Republic of China

## Abstract

The heterojunction effects of TiO_2 _nanotubes on photoconductive characteristics were investigated. For ITO/TiO_2_/Si diodes, the photocurrent is controlled either by the TiO_2_/Si heterojunction (p-n junction) or the ITO-TiO_2 _heterojunction (Schottky contact). In the short circuit (approximately 0 V) condition, the TiO_2_-Si heterojunction dominates the photocarrier transportation direction due to its larger space-charge region and potential gradient. The detailed transition process of the photocarrier direction was investigated with a time-dependent photoresponse study. The results showed that the diode transitioned from TiO_2_-Si heterojunction-controlled to ITO-TiO_2 _heterojunction-controlled as we applied biases from approximately 0 to -1 V on the ITO electrode.

## Background

In recent years, nanostructure materials have attracted much interest due to their remarkable physical and chemical properties. Among these nanostructure materials, TiO_2 _nanostructures have emerged as one of the most promising materials for optoelectronic devices because of the variety of growth methods and their high melting point (1,855°C), chemical inertness, physical stability, indirect band gap (3.2 eV), high photoconversion efficiency, and photostability. Based on its excellent optical properties, TiO_2 _has been utilized for many applications, such as photoelectrochemical water splitting [1], photoelectrochemical generation of hydrogen [2], dye-sensitized solar cells [3], and photocatalysis [4].

Unfortunately, the inherent high band gap of 3.2 eV limits the optical application of TiO_2_. Therefore, most of the research efforts have been focused on modifying the material properties with the hope of enhancing the absorbability of TiO_2 _to extend from the ultraviolet (UV) region to the visible region through a doping process [5,6]. However, in addition to modifying material properties, it is essential to understand and pay special attention to heterojunctions in the study of traditional semiconductors because the heterojunction effect determines a device's ultimate performance. Furthermore, when nanoscale materials are utilized, the heterojunction effects are magnified and become even more critical. For heterojunction semiconductor devices, the type of contact determines the carrier transportation direction, and the space-charge region and the potential gradient of a junction determine the magnitude of the photocurrent as the devices are illuminated by a light source [7,8]. Therefore, apart from the modification of the intrinsic material, heterojunction studies of devices or sensors under UV light illumination should not be neglected. Wang et al. reported that N-TiO_2_/C heterojunctions could increase absorption in the visible light region and exhibit a higher photocatalytic activity than pure TiO_2 _[9]. Zhang et al. reported that the heterojunction of Bi_2_MoO_6_/TiO_2 _shows effective separation of photo-generated carriers driven by the photo-induced potential difference generated at the Bi_2_MoO_6_/TiO_2 _heterojunction interfaces [10]. Lee et al. reported that a TiO_2_/water solid-liquid heterojunction exhibits a high photosensitivity, excellent spectral selectivity, linear variations in photocurrent, and fast responses with ultraviolet [11]. As mentioned above, the study of TiO_2 _heterojunctions has focused on single heterojunctions. However, there are two heterojunctions in many devices, such as solar cells [12]. Therefore, more studies in this field are necessary, especially in nanostructure systems.

In this research, we employed an anodic aluminum oxide (AAO) template and atomic layer deposition (ALD) nanotechnology to prepare TiO_2 _nanotube arrays. A thin ITO electrode was deposited on top of the TiO_2 _to form a Schottky contact [13], and the heterojunction effects on the photoconductive characteristics of TiO_2 _nanotubes under forward and reverse biases were investigated. To explain the change of the carrier transportation direction, we discussed the mechanism using an energy band diagram.

## Methods

To fabricate TiO_2 _nanostructures, first, AAO was prepared on p-type (100) silicon substrates with two-stage anodization. The detailed fabrication process for the AAO was presented elsewhere [14]. The diameters of the AAO pores are 70 nm. In particular, the AAO barrier has been removed after the pore widening treatment so that the deposited TiO_2 _nanostructure can adhere to the substrate after the removal of the AAO template.

To deposit the TiO_2 _nanostructure by ALD, Si substrates with the AAO templates were first placed into a quartz tube reactor with the operating environment maintained at 1.6 × 10^-1 ^Torr and 400°C. The precursors of TiCl_4 _and H_2_O, kept separately in a canister at 30 ± 1°C and 25 ± 1°C respectively, were used as Ti and O sources, respectively. Pure Ar gas (99.999%) was used as a carrier gas and purge gas. To prepare TiO_2 _nanotube arrays, a 300-cycle deposition parameter was introduced. Each deposition cycle consisted of eight steps, which included TiCl_4 _reactant, pump-down; Ar purge, pump-down; H_2_O reactant, pump-down; and Ar purge and pump-down. Typical pulse times for the TiCl_4 _and H_2_O precursors were 1 s, and the purge time was 3 s. To remove the residual reactants and by-products efficiently, the pump-down process was added after each step. Then, with mechanical polishing, the TiO_2 _film on the top surface of AAO was removed. Finally, the AAO template was selectively removed by a 0.1 wt% sodium hydroxide (aqueous) solution, and TiO_2 _nanotube arrays were fabricated on the Si substrate.

Highly ordered self-aligned TiO_2 _nanotubes can be fabricated using the template of (AAO) on p-type (100) Si substrates and the ALD technique [15]. To prepare TiO_2 _nanotube arrays, a 300-cycle deposition parameter was adopted. An ITO film (450 nm) and Al film were chosen as the electrode and the back electrode for ohmic contact and were deposited using an e-gun evaporation system and a thermal evaporation coater, respectively. The current characteristics of the specimens were recorded by a Keithley 2400 sourcemeter (Keithley Instruments Inc., Cleveland, Ohio). Due to the measuring requirement, a small voltage of approximately 10^-6 ^V is applied automatically by the Keithley 2400 sourcemeter when the equipment is set up to 0 V bias. Nevertheless, it is regarded as a short circuit (approximately 0 V) condition because 10^-6 ^V can be considered negligible. To perform the study, the photoresponse was measured under UV illumination of approximately 21 mW/cm^2 ^(λ = 365 nm) in air at room temperature. A field-emission scanning electron microscope (FESEM JSM-6500 F, JEOL Ltd., Tokyo, Japan) and transmission electron microscopy (TEM) were utilized to examine the morphology of the TiO_2 _nanotube arrays.

## Results and discussion

**Figure 1:**
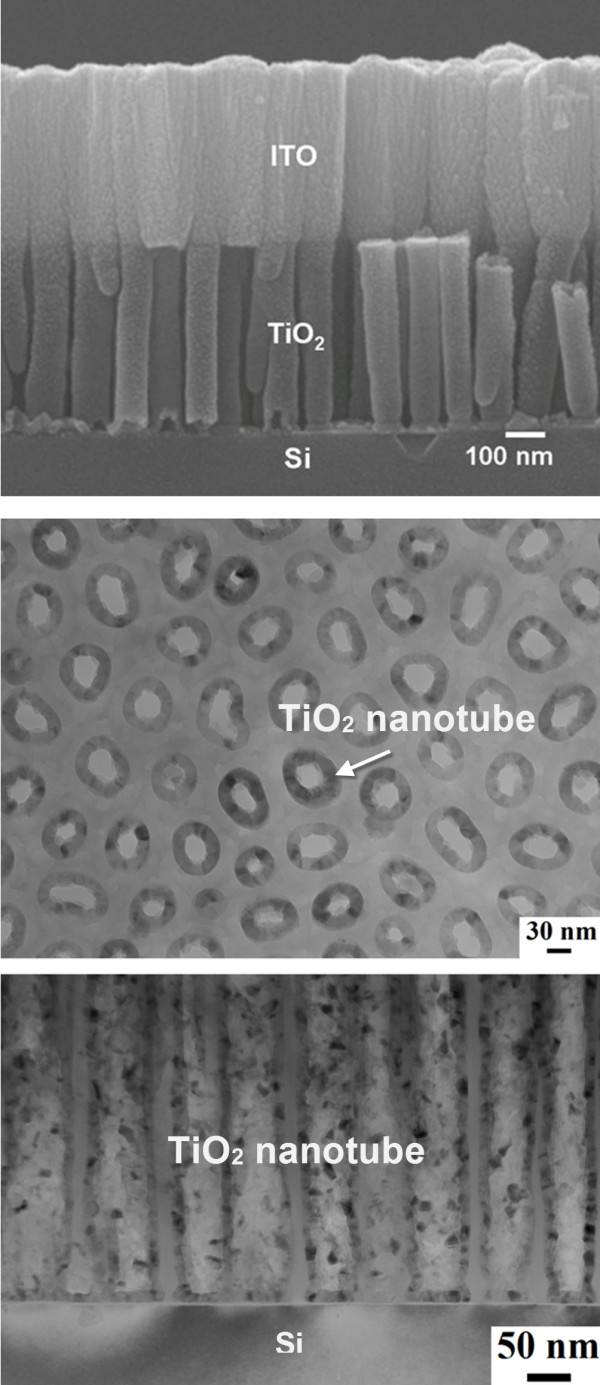
**Cross-sectional SEM and TEM images and plan view of nanotube arrays**. *Top *image, cross-sectional SEM image showing the fabricated TiO_2 _nanotubes with an ITO electrode on the top surface. *Center *image, plan view TEM images of TiO2 nanotube arrays. *Bottom *image, cross-sectional TEM images for the TiO2 nanotube arrays.

To understand the ITO/TiO_2_/Si diode structure, we used scanning electron microscopy (SEM) and TEM to analyze the structure. Figure 1a shows the cross-sectional SEM image of TiO_2 _nanotubes with an ITO electrode deposited on top. No residual AAO template was observed. The nanotubes are perpendicular to the Si substrate and are in good contact with the ITO. The height of the TiO_2 _nanotube arrays is 485 nm. The plan view TEM image of the TiO_2 _nanotubes is shown in Figure 1b. The average wall thickness of the nanotubes is measured to be 17.3 nm, and the thickness appears to be very uniform. Figure 1c presents the cross-sectional TEM image for the TiO_2 _nanotubes on a Si substrate. The average diameter of TiO_2 _nanotubes is 70 nm.

**Figure 2:**
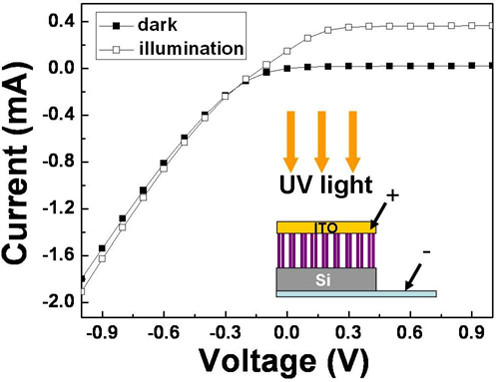
***I-V *characteristics of the ITO/TiO_2_/Si diode with a positive bias on the ITO electrode**. The embedded drawing shows the experimental setup.

*I-V *characteristics of the ITO/TiO_2_/Si diode are presented in Figure 2. Embedded in the graph is a measured schematic diagram which shows that a positive bias is applied to the ITO electrode. When a positive bias is applied to the ITO, the ITO-TiO_2 _heterojunction is under a forward bias; nevertheless, the TiO_2_-Si heterojunction is under a reverse bias. The rectified current characteristic of the photodiode is observed in the dark environment, which is the same as the traditional *I-V *characteristic of a p-n junction at a reverse bias [16]. Therefore, the reverse-biased rectification properties highlighted the fact that the TiO_2_-Si heterojunction at a reverse bias dominated the performance of carrier transportation. In Figure 2, we also observed that when UV illumination was applied, the photocurrent exhibits a flattening in the *I-V *characteristics above 0.3 V. The flattening of the curve occurs because the space-charge region of the TiO_2_/Si heterojunction may extend to the entire TiO_2 _nanotube array. Therefore, the photocurrent reaches saturation when the voltage is 0.3 V. In contrast, the photocurrent gradually increases with the increase in the negative biases. That is, the ITO-TiO_2 _junction was under reverse bias. With increasing negative bias, the ITO-TiO_2 _junction may become larger, which may contribute to the photocurrent. A detailed discussion will be provided later in the text.

**Figure 3:**
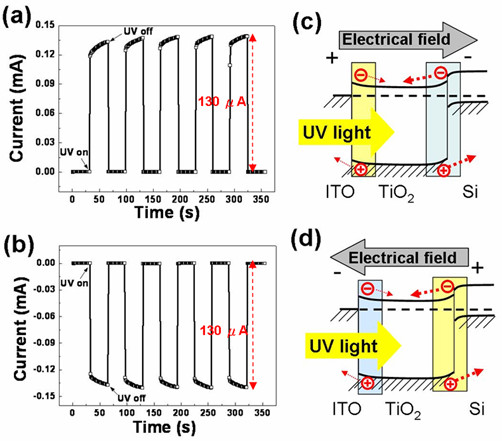
**Short circuit current as a function of time under on/off UV illumination cycles**. The short circuit current as a function of time under on/off UV illumination cycles when a positive bias is applied to the ITO electrode (**a**) and when a negative bias is applied (**b**). Figures (**c**) and (**d**) are the corresponding energy band diagrams with respect to (a) and (b).

Figure 3a, b shows the short circuit current under on/off UV illumination cycles as a function of time when ITO is connected to the positive electrode and Si is attached to the negative electrode. Because the current is recorded once per second and it allows enough time for the photoreaction to stabilize [17,18], the short circuit (approximately 0 V) current at each UV on/off cycle is steady and reproducible. To further explain the short circuit photoresponses, the energy band diagrams for the heterojunctions are introduced to illustrate the photocarrier transportation directions. A detailed discussion of the energy band diagrams was presented in a prior study [19]. Under UV illumination, photocurrents with opposing directions were generated from two space-charge regions located at the TiO_2_-Si heterojunction (p-n junction) and the ITO-TiO_2 _heterojunction (Schottky contact). When the ITO electrode is connected to the positive electrode, the positive photocurrent (0.13 mA) shown in Figure 3a has the same direction as the electric field, as shown by the thick dotted arrows in Figure 3c. When the negative electrode is connected to the ITO, the directions of the photocurrent (-0.13 mA) and the electric field are opposite, as shown in Figure 3b, d. Both cases indicated that the TiO_2_-Si heterojunction dominated the photocurrent transportation direction, as shown in Figure 3d. In addition, the same absolute magnitude of the photocurrent also indicated that both cases come from the same generating source. Again, we confirm that the TiO_2_-Si heterojunction, owing to its wider space-charge region and larger potential gradient, dominated the photocurrent transportation direction and photocurrent magnitude, in agreement with the results of the *I-V *characteristics in Figure 2.

**Figure 4:**
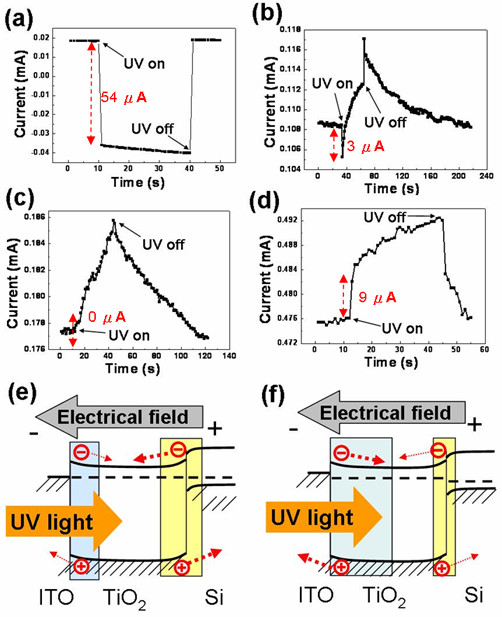
**Time-dependent current for the ITO/TiO_2_/Si diode under UV on/off illumination**. Time-dependent current for the ITO/TiO_2_/Si diode under UV on/off illumination when the following biases are applied to the ITO electrode: (**a**) -0.1 V, (**b**) -0.4 V, (**c**) -0.6 V, and (**d**) -1 V. (**e**) and (**f**) are the energy band diagrams in the short circuit condition and at a negative bias, respectively.

Figure 4a, b, c, d shows the time-dependent current for the ITO/TiO_2_/Si diode under UV on/off illumination when negative biases from approximately 0 to -1 V were applied to the ITO electrode. As the UV light source was turned on, the magnitude and the direction of the current changed from 0.018 to -0.036 mA at -0.1 V. As mentioned in the short circuit (approximately 0 V) condition above, this phenomenon means that the TiO_2_-Si heterojunction still controls the photocarrier transportation direction, even at -0.1 V bias. According to the classical knowledge in semiconductors [20], it is known that when a negative bias is applied to the ITO electrode, the TiO_2_-Si heterojunction and the ITO-TiO_2 _heterojunction are under a forward bias and a reverse bias, respectively. It is also known that the width of the space-charge region and the potential gradient are enlarged at a larger reverse bias, whereas they are narrowed at a larger forward bias, as shown in Figure 4e, f. Therefore, for short circuit -0.1 and -0.4 V bias, the net photocurrent change from 'off' to 'on' UV illumination was decreased from 0.13 to 0.054 mA and to 3 mA due to the narrowed space-charge region and the potential gradient at the TiO_2_-Si heterojunction under a forward bias, as shown in Figure 4a, b. The decrease in the current at the moment from off to on UV illumination means that the TiO_2_-Si heterojunction is still dominant in those cases. In addition, the current increased after UV was shut off. This phenomenon may be attributed to the escape of photocarriers trapped by the defects in the heterojunctions. With larger biases, trapped photocarriers were discharged more easily in a short time.

Although the fast photoresponse can be observed at the UV on/off moment, the gradual increase and the slow recovery of the photocurrent under UV on/off illumination were recorded when the bias was -0.4 V. However, when a -0.6 V bias was applied, no net photocurrent magnitude was observed under UV illumination, except for the gradual increase and slow recovery of the photocurrent, as shown in Figure 4c. This phenomenon means that we have reached the transition point because the photocurrent generated from the ITO-TiO_2 _heterojunction was enhanced at an increased reverse bias, whereas the current generated from the TiO_2_-Si heterojunction was reduced at an increased forward bias. The two currents reached equal magnitudes at the transition point, thus canceling each other out. After the bias was enlarged to -1 V, the current was increased from 0.476 to 0.485 mA under UV illumination, as shown in Figure 4d. The photocurrent originating from the ITO-TiO_2 _heterojunction was large enough to counteract the effects of the photocurrent resulting from the TiO_2_-Si heterojunction, finally governing the photocarrier transportation direction. However, the gradual increase and slow recovery in the photocurrent were still observed. Nevertheless, the important finding of our study is that we have observed that the transition point of the photocurrent transportation direction changes from TiO_2_-Si heterojunction-controlled to ITO-TiO_2 _heterojunction-controlled and from short circuit to -1 V.

From the above results, we speculate that the photocarrier transportation may be dominated by the space-charge region and the potential gradient of the heterojunctions. However, the area ratio of the heterojunction of TiO_2_-Si to ITO-TiO_2 _should be considered. We suppose that more heterojunction area would contribute more photocarriers when UV illumination is used. The photocarriers move in opposite direction for the TiO_2_-Si and ITO-TiO_2 _heterojunctions. From Figure 1b, c, the heterojunction area ratio can be calculated by the following equation: $\frac{\text{junction}\;\text{area}\;\text{of}\;\text{Ti}{\text{O}}_{\text{2}}\text{ - Si}}{\text{junction}\;\text{area}\;\text{of}\;\text{ITO - Ti}{\text{O}}_{\text{2}}}=\frac{\pi \times r_{\text{Ti}{\text{O}}_{\text{2}}}^{\text{2}}}{\pi \times r_{\text{Ti}{\text{O}}_{\text{2}}}^{2}-\pi {\left[\left(r_{\text{Ti}{\text{O}}_{\text{2}}}-t_{\text{Ti}{\text{O}}_{\text{2}}}\right)\right]}^{2}}=4.1$

where *r*_TiO2 _is the dimension of the TiO_2 _nanotubes and *t*_TiO2 _is the wall thickness of the TiO_2 _nanotubes. The voltage of the transition point is -0.6 V when the heterojunction area ratio is 4.1. The voltage at the transition point may decrease as the junction area ratio decreases. Nevertheless, further studies are required to clarify this point.

## Conclusions

In summary, we studied the heterojunction effects on the UV photoresponse of TiO_2 _nanotubes fabricated by ALD on a Si substrate using ITO as the electrode. In the short circuit (approximately 0 V) condition, photocurrents of 0.13 and -0.13 mA were measured when the positive and the negative electrodes were connected to the ITO, respectively. When a negative bias was applied, the net photocurrent changed from off to on and UV illumination decreased from 0.13 mA to 3 μA as the negative bias was decreased from short circuit (approximately 0) to -0.4 V. The photocurrent reached a transition point when -0.6 V was applied, which is where current generated by the ITO-TiO_2 _heterojunction equals the current generated by the TiO_2_-Si heterojunction. These intriguing results may be attributed to the depletion regions in the ITO-TiO_2 _and Si-TiO_2 _heterojunctions. More studies are needed to clarify which heterojunction dominates the photocurrent.

## References

[B1] Park JH Kim S Bard AJ Novel carbon-doped TiO_2 _nanotube arrays with high aspect ratios for efficient solar water splitting Nano Lett 2006 6 24 28 10.1021/nl051807y 16402781

[B2] Rao NN Dube S Photoelectrochemical generation of hydrogen using organic pollutants in water as sacrificial electron donors Int J Hydrogen Energy 1996 21 95 98 10.1016/0360-3199(95)00045-3

[B3] Grätzel M Photoelectrochemical cells Nature 2001 414 338 344 10.1038/35104607 11713540

[B4] Liu Z Sun DD Guo P Leckie JO An efficient bicomponent TiO_2_/SnO_2 _nanofiber photocatalyst fabricated by electrospinning with a side-by-side dual spinneret method Nano Lett 2007 7 1081 1085 10.1021/nl061898e 17425281

[B5] Park JH Park OO Kim S Photoelectrochemical water splitting at titanium dioxide nanotubes coated with tungsten trioxide Appl Phys Lett 2006 89 163106 163109 10.1063/1.2357878

[B6] Umebayashi T Yamaki T Itoh H Asai K Band gap narrowing of titanium dioxide by sulfur doping Appl Phys Lett 2002 81 454 457 10.1063/1.1493647

[B7] Agrawal R Kumar P Ghosh S Mahapatro AK Thickness dependence of space charge limited current and injection limited current in organic molecular semiconductors Appl Phys Lett 2008 93 073311 073314 10.1063/1.2974084

[B8] Alivov YI Van Nostrand JE Look DC Chukichev MV Ataev BM Observation of 430 nm electroluminescence from ZnO/GaN heterojunction light-emitting diodes Appl Phys Lett 2003 83 2943 2946 10.1063/1.1615308

[B9] Wang DH Jia L Wu XL Lu LQ Xu AW One-step hydrothermal synthesis of N-doped TiO_2_/C nanocomposites with high visible light photocatalytic activity Nanoscale 2012 4 576 584 10.1039/c1nr11353d 22143193

[B10] Zhang M Shao C Mu J Zhang Z Guo Z Zhang P Lin Y One-dimensional Bi_2_MoO_6_/TiO_2 _hierarchical heterostructures with enhanced photocatalytic activity Cryst Eng Comm 2012 14 605 612

[B11] Lee WJ Hon MH An ultraviolet photo-detector based on TiO_2_/water solid-liquid heterojunction Appl Phys Lett 2011 99 2511021 2511023

[B12] Zhang TW Ho CC Tu YC Tu GY Wang LY Su WF Correlating interface heterostructure, charge recombination, and device efficiency of poly (3-hexyl thiophene)/TiO_2 _nanorod solar cell Langmuir 2011 27 15255 15260 10.1021/la203533u 22050188

[B13] Dai W Wang X Liu P Xu Y Li G Fu X Effects of electron transfer between TiO_2 _films and conducting substrates on the photocatalytic oxidation of organic pollutants J Phys Chem B 2006 110 13470 13476 10.1021/jp061483h 16821872

[B14] Yang CJ Wang SM Liang SW Chang YH Chen C Shieh JM Low-temperature growth of ZnO nanorods in anodic aluminum oxide on Si substrate by atomic layer deposition Appl Phys Lett 2007 90 033104 033107 10.1063/1.2431786

[B15] Liu CM Chen C Cheng HE Growth mechanism of TiO2 nanotube arrays in nanopores of anodic aluminum oxide on Si substrates by atomic layer deposition J Electrochem Soc 2011 158 K58 K63 10.1149/1.3528939

[B16] Neamen DA Chapter 7 Semiconductor Physics and Devices 1997 2 Chicago: McGraw-Hill

[B17] Kongkanand A Tvrdy K Takechi K Kuno M Kamat PV Quantum dot solar cells. tuning photoresponse through size and shape control of CdSe-TiO_2 _architecture J Am Chem Soc 2008 130 4007 4015 10.1021/ja0782706 18311974

[B18] Lin YY Chen CW Yen WC Su WF Ku CH Wu JJ Near-ultraviolet photodetector based on hybrid polymer/zinc oxide nanorods by low-temperature solution processes Appl Phys Lett 2008 92 233301 233304 10.1063/1.2940594

[B19] Chang YH Liu CM Tseng YC Chen C Chen CC Chen HE Direct probe of heterojunction effects upon photoconductive properties of TiO_2 _nanotubes fabricated by atomic layer deposition Nanotechnology 2010 21 225602 225609 10.1088/0957-4484/21/22/225602 20453279

[B20] Neamen DA Chapter 6 Semiconductor Physics and Devices 1997 2 Chicago: MacGraw-Hill

